# Inhibitory STDP improves temporal processing in disynaptic circuits

**DOI:** 10.1186/1471-2202-14-S1-P134

**Published:** 2013-07-08

**Authors:** Florence I Kleberg, Matthieu Gilson, Tomoki Fukai

**Affiliations:** 1 RIKEN BSI, Wakoshi, Saitama, 351-0109, Japan; 2 Universitat Pompeu Fabra, Barcelona, E-08018, Spain

## 

Spike trains with temporal correlations have been related to cognitive and sensory processing [1], and extraction of correlation information may therefore be of great importance to neuronal computations. Synaptic plasticity rules such as Spike-Timing Dependent Plasticity (STDP) allow selective increases of synaptic weights that receive correlated spike input, allowing the neuron to detect correlation in its input [2,3]. Though this has been shown in excitatory synapses, only few studies have addressed the functional properties of STDP in inhibitory synapses [4,5].

It is known that excitatory and inhibitory activities are typically balanced [6], and maintenance of a 'detailed balance' within signaling pathways has been shown to achieve useful gating properties [7]. Therefore, combining plasticity mechanisms in excitatory and inhibitory weights in the same model could provide insight in how such a balance can arise, and what possible functions inhibitory synaptic plasticity can fulfill in the presence of input spike trains with varying statistics.

**Figure 1:**
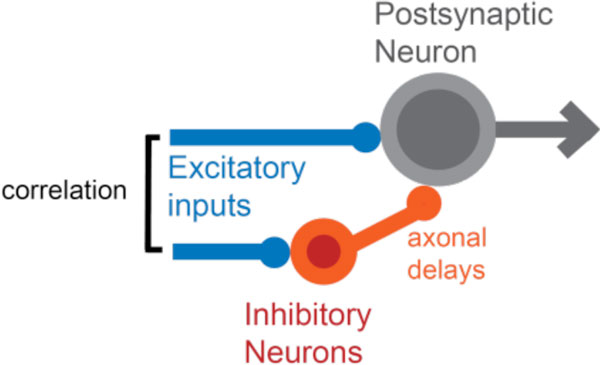
**A canonical disynaptic feed-forward circuit considered in our study**. The inhibitory input is modeled as a delayed spike train, correlated with the excitatory input.

In this study, we use a Leaky-Integrate-And-Fire neuron with inputs that model a canonical disynaptic feedforward-inhibitory circuit (Figure 1). We show analytically and computationally that this detailed balance can be achieved by STDP in inhibitory and excitatory synapses, depending on the characteristics of the inhibitory STDP learning window. Our results further indicate that in such a configuration, the postsynaptic response to excitatory inputs is rendered more reliable in time, and that there is an optimal inhibitory delay for which this improvement is effective. Moreover, we extend our model to explicitly simulate the fast-spiking inhibitory neurons, and confirm that inhibitory neurons are recruited to different sources of correlation by STDP, and that inhibitory synapses subsequently follow the same scenario as in the single-neuron model.

Our findings provide insight in the role of inhibitory STDP, in maintaining detailed balance and enhancing transmission efficacy in disynaptic neural circuits.
